# Assessment of reporting practices and reproducibility potential of a cohort of published studies in computational knee biomechanics

**DOI:** 10.1002/jor.25358

**Published:** 2022-05-22

**Authors:** Jason P. Halloran, Neda Abdollahi Nohouji, Mhd A. Hafez, Thor F. Besier, Snehal K. Chokhandre, Shady Elmasry, Donald R. Hume, Carl W. Imhauser, Nynke B. Rooks, Marco T. Y. Schneider, Ariel Schwartz, Kevin B. Shelburne, William Zaylor, Ahmet Erdemir

**Affiliations:** ^1^ Institute for Shock Physics, Applied Sciences Laboratory Washington State University Spokane Washington USA; ^2^ Center for Human Machine Systems Cleveland State University Cleveland Ohio USA; ^3^ Department of Mechanical Engineering Cleveland State University Cleveland Ohio USA; ^4^ Department of Biomedical Engineering, Lerner Research Institute Cleveland Clinic Cleveland Ohio USA; ^5^ Department of Civil Engineering Cleveland State University Cleveland Ohio USA; ^6^ Auckland Bioengineering Institute University of Auckland Auckland New Zealand; ^7^ Department of Engineering Science, Faculty of Engineering University of Auckland Auckland New Zealand; ^8^ Computational Biomodeling (CoBi) Core, Lerner Research Institute Cleveland Clinic Cleveland Ohio USA; ^9^ Department of Biomechanics Hospital for Special Surgery New York New York USA; ^10^ Department of Mechanical and Materials Engineering University of Denver Denver Colorado USA; ^11^ Center for Orthopaedic Biomechanics University of Denver Denver Colorado USA

**Keywords:** biomechanics, finite element analysis, knee model, reproducibility, rigid‐body analysis

## Abstract

Reproducible research serves as a pillar of the scientific method and is a foundation for scientific advancement. However, estimates for irreproducibility of preclinical science range from 75% to 90%. The importance of reproducible science has not been assessed in the context of mechanics‐based modeling of human joints such as the knee, despite this being an area that has seen dramatic growth. Framed in the context of five experienced teams currently documenting knee modeling procedures, the aim of this study was to evaluate reporting and the perceived potential for reproducibility across studies the teams viewed as important contributions to the literature. A cohort of studies was selected by polling, which resulted in an assessment of nine studies as opposed to a broader analysis across the literature. Using a published checklist for reporting of modeling features, the cohort was evaluated for both “reporting” and their potential to be “reproduced,” which was delineated into six major modeling categories and three subcategories. Logistic regression analysis revealed that for individual modeling categories, the proportion of “reported” occurrences ranged from 0.31, 95% confidence interval (CI) [0.23, 0.41] to 0.77, 95% CI: [0.68, 0.86]. The proportion of whether a category was perceived as “reproducible” ranged from 0.22, 95% CI: [0.15, 0.31] to 0.44, 95% CI: [0.35, 0.55]. The relatively low ratios highlight an opportunity to improve reporting and reproducibility of knee modeling studies. Ongoing efforts, including our findings, contribute to a dialogue that facilitates adoption of practices that provide both credibility and translation possibilities.

## 1 INTRODUCTION

**Figure 1:**
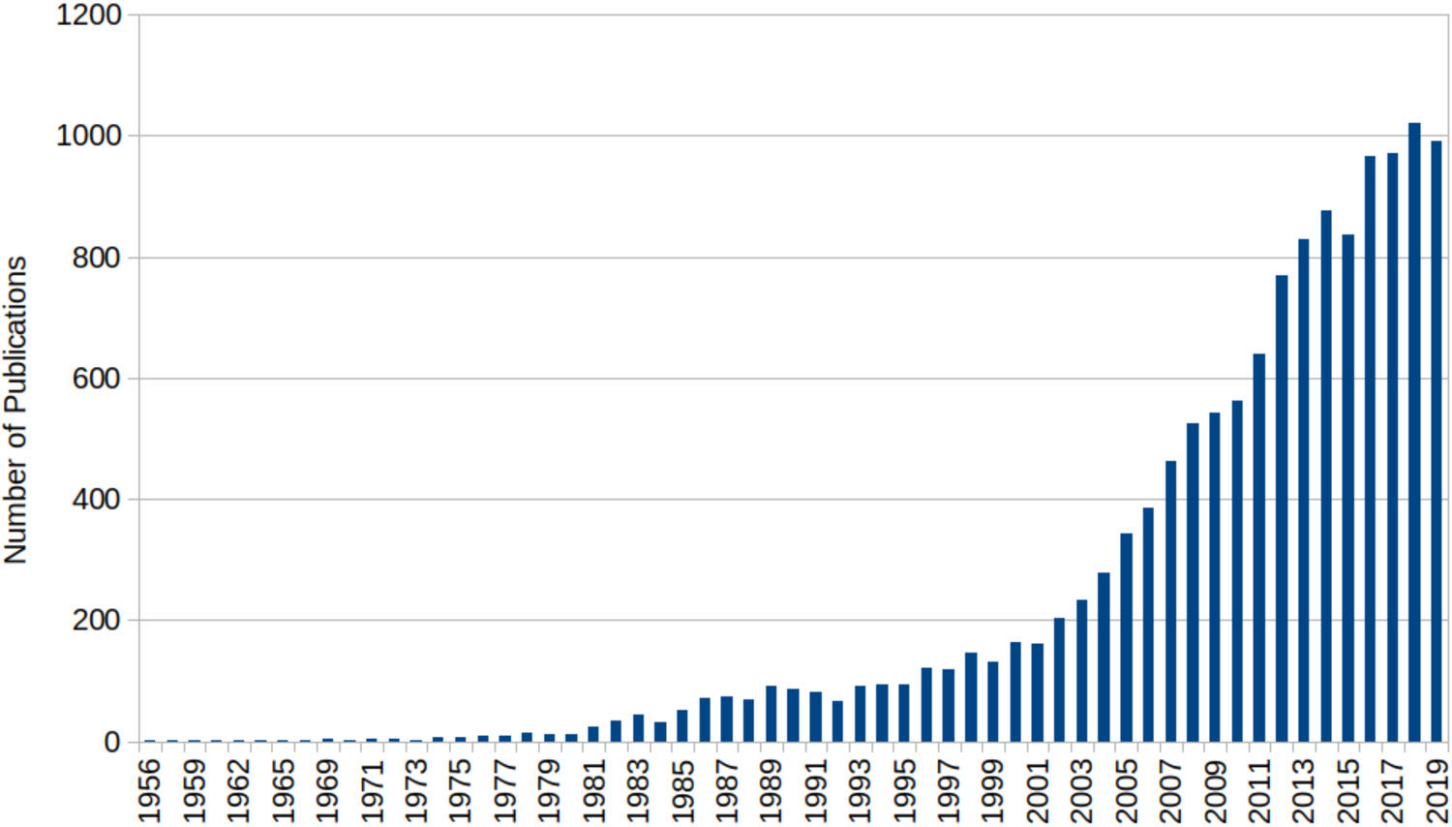
The number of publications per year related to computational modeling of the knee have increased dramatically over previous decades. References were found using PubMed and the search string “knee” AND (“computational” OR “rigid body” OR “finite element”). [Color figure can be viewed at wileyonlinelibrary.com]

Reproducibility is a key component of the scientific method and is a foundation for scientific advancement. Yet, recent studies indicate that 75%–90%^1^, ^2^, ^3^, ^4^ of preclinical research is not reproducible. Irreproducibility is an important problem because it not only hinders discovery of new medical treatments but also erodes confidence, for both the public and expert practitioners, in preclinical scientific findings. Preclinical computational research faces unique reproducibility challenges due to its developmental and exploratory nature, often foregoing consideration of reuse and reproducibility in favor of addressing the specific research question at hand. Despite the importance of reproducible science, this topic has not been addressed in the field of mechanics‐based modeling of human joints such as the knee. Since applications of computational modeling in this area have grown exponentially over the past two decades (Figure 1), assessing reproducibility related to commonly used modeling approaches is warranted. Understanding the current state of reproducibility in computational modeling of the knee is also critical because of the potential of this technology to impact clinical practice. For example, modeling may help address topics ranging from understanding mechanisms of traumatic knee injury, such as rupture of the anterior cruciate ligament, to revealing mechanical factors associated with onset and progression of osteoarthritis.^5^, ^6^, ^7^, ^8^

**Figure 2:**
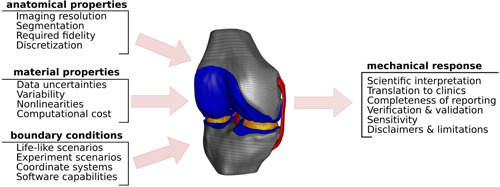
Mechanics‐based modeling of the knee (and other joints) requires consideration of anatomy, material properties, and boundary conditions. Within each of these categories numerous modeling decisions are required. A snapshot of the items that were reviewed for their reporting and potential for reproducibility is listed. The predicted mechanical response of the knee model is subject to additional considerations. [Color figure can be viewed at wileyonlinelibrary.com]

Computational modeling of the knee requires expertise in numerous areas. Common steps in the modeling workflow include developing anatomical representations of the bones and soft tissues, assigning material properties, applying loads and boundary conditions, and interpreting model predictions (Figure 2). Each step includes numerous requirements and approaches such as discretizing the anatomy, assessing variability in material response, and applying loads and boundary conditions. These processes (and subprocesses) are often lab and modeler‐dependent, and rely on the chosen computational methods, and the strategies through which modeling decisions are made may impact the ability to reproduce such studies. Many of these challenges may be partially addressed through adequate reporting and assessment practices.^9^, ^10^, ^11^, ^12^

There are several challenges in developing reproducible knee‐specific mechanical models, which can be directly related to the modeling process (Figure 2). First is the high degree of intersubject variability in the anatomy, in material response of the involved tissues, and in loading of the knee and its structures. Second is the complex model creation processes that necessitate mathematically defining such features. Third is varied modeling approaches both within and across research groups. Finally, limited experimental data hinder analysis of large cohorts of knees, which results in focused studies using relatively few specimens whose conclusions may not be generalizable.^13^, ^14^, ^15^, ^16^ The potential for both patient‐specific and population‐based studies to support patient care may be bolstered through concerted efforts to assess the current state of reproducibility.

The proliferation of knee modeling and the diversity of these efforts warrants the assessment of the state of reproducibility in computational modeling of this joint. In this study, five teams active in documenting and disseminating mechanics‐based knee modeling procedures (Erdemir et al., 10; “SimTK: Reproducibility in simulation‐based prediction of natural knee mechanics: Project Home, 17”) evaluated a representative selection of previously published studies in computational modeling of the knee to address three research questions: (1) Are important components of the modeling workflow reported?; (2) What is the perceived potential for reproducibility of these components?; and (3) Did the perception of whether each modeling component was reported and whether it was reproducible differ? Addressing these research questions will identify unique reproducibility challenges facing mechanics‐based modeling of the knee and, therefore, will contribute to the ongoing dialogue surrounding reproducible science.

## 2 METHODS

### 2.1 Reviewers

This study was undertaken as part of an ongoing project to openly document and disseminate modeling practices in the knee.^10^, ^17^ The following five teams comprised the reviewers in this study; each team has an established record in knee modeling.^9^, ^15^, ^18^, ^19^, ^20^, ^21^, ^22^, ^23^ Together, the 14 authors have more than 160 years of combined experience conducting finite element analysis or rigid‐body motion simulations.

1. Department of Biomedical Engineering, Cleveland Clinic (CC).
2. Department of Biomechanics, Hospital for Special Surgery (HSS).
3. The Auckland Bioengineering Institute at the University of Auckland (UoA).
4. The Center for Orthopaedic Biomechanics at the University of Denver (DU).
5. The Applied Sciences Laboratory at Washington State University in collaboration with the Center for Human Machine Systems at Cleveland State University (WSU/CSU).

### 2.2 Selection of the representative cohort

A cohort of studies was selected by polling the five participating research teams. Each team independently submitted a list of at least 10 publications they viewed as valuable to their work in computational biomechanics of the knee. Each team could submit publications that included authors of this manuscript, but they could not include studies that originated from their own group. Studies needed to include the tibiofemoral joint; inclusion of the patellofemoral articulation was not required. The model formulation, for example, rigid‐body or finite element, was not dictated, leaving each research team to identify studies they viewed as important contributions to the literature. Studies were also not limited to any particular publication timeframe. Each team provided their respective lists in the summer of 2019. Due to the significant undertaking of assessing each individual study twice by different research teams (see Section 2.4), the number of included studies was limited to a representative cohort. To determine the representative cohort for assessment, at least three out of five teams were required to have included a given publication in their respective list. This criteria resulted in the selection of nine studies (Table 1).^9^, ^13^, ^15^, ^20^, ^24^, ^25^, ^26^, ^27^, ^28^

**Table 1:** List of the nine publications used to evaluate reporting practices and the perceived potential for reproducibility.

	Publication
1	Blankevoort L, Huiskes R. *J Biomech*. 1996 Jul;29(7):955–961. doi:10.1016/0021-9290(95)00149-2
2	Dhaher YY, et al. *J Biomech*. 2010 Dec 1;43(16):3118–25. doi:10.1016/j.jbiomech.2010.08.005
3	Donahue TL, et al. *J Biomech Eng*. 2002 Jun;124(3):273–80. doi:10.1115/1.1470171
4	Erdemir A. *J Knee Surg*. 2016 Feb;29(2). doi:10.1055/s-0035-1564600
5	Harris MD, et al. *J Biomech Eng*. 2016 Aug 1;138(8). doi:10.1115/1.4033882
6	Kiapour A, et al. *J Biomech Eng*. 2014 Jan;136(1):01102. doi:10.1115/1.4025692
7	Kia M, et al. *J Biomech Eng*. 2016 May;138(5):051010. doi:10.1115/1.4032850
8	Naghibi Beidokhti H, et al. *J Biomech*. 2017 Dec 8;65:1–11. doi:10.1016/j.jbiomech.2017.08.030
9	Peña E, et al. *J Biomech*. 2006;39(9):1686–1701. Epub 2005 Jul 1. doi:10.1016/j.jbiomech.2005.04.030

### 2.3 Evaluation criteria and outcome measures

**Figure 3:**
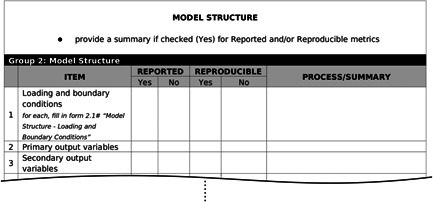
Snapshot of the “model structure” category's checklist including the first three model components. All reviews consisted of filling in the checklist, which included entries for all major categories and subcategories (e.g., specific forms for each loading and boundary condition). With the addition of the “reproducible” metric the checklist was based on a previously published study on reporting of finite element simulations in biomechanics.^10^

Assessment of reporting (Research Question 1) and perceived potential for reproducibility (Research Question 2) followed a modified version of a published checklist for reporting of finite element studies in biomechanics.^12^ In addition to whether a model feature and procedure were reported, the primary modification over the published checklist included an evaluation of “reproducibility potential” (Figure 3). This subjective evaluation criteria allowed the reviewers to consider whether a particular modeling feature could be reproduced. The major modeling categories and subcategories are listed below while the checklists completed by each team are available in a download package (see “https://doi.org/10.18735/w9qc-9h39”).

The reviewers provided a binary (yes or no) assessment of their perception of whether each model feature was reported, whether it was reproducible and explained their decisions (Figure 3). In total, six major categories of modeling features were evaluated for reporting and five of these categories were also assessed for perceived reproducibility. Model Identification was the lone category excluded from the assessment of perceived reproducibility because items such as naming, assigning keywords, etc. can be arbitrary and do not impact potential reproducibility. The Model Structure category included three subcategories, which were analyzed separately (see Section 2.5) using a checklist (snapshot in Figure 3). The six major categories and subcategories, including an abbreviated list of each category's components, are summarized below:

1. **Model Identification**
  (a) Model name, keywords, version, physiological domain, and so on.

2. **Model Structure**
  (a) Primary outputs, secondary outputs, source of anatomy, imaging modality, and resolution.

2.1 **Loading and boundary conditions**
  (a) Type (e.g., reaction, displacement, etc.), process to obtain, region, and so on.

2.2 **Subcomponents (subsidiary models)**
  (a) Name, geometric representation, mesh and/or process to represent geometry, constitutive formulation, and so on.

2.3 **Interactions**
  (a) Interacting components and interaction type, formulation, and properties.

3. **Simulation structure**
  (a) Simulation software, version, solution strategy, and so on.

4. **Verification**
  (a) Method of verification, correctness of formulation, comparison with known solutions, and so on.

5. **Validation**
  (a) Validation process, validated output, overall modeling assumptions, and so on.

6. **Availability**
  (a) Contact information, licensing, website for downloads, and so on.

### 2.4 Review procedure

Each of the nine studies was independently reviewed twice. To realize the 18 total reviews the CSU/WSU team reviewed two manuscripts while the remaining teams reviewed four each. Manuscripts were assigned at random to each team. Three of the nine reviewed studies included coauthors of this manuscript. If a research team was randomly assigned to one of their own studies, a new set of random assignments was initiated until this was not the case. Reviews were submitted to the WSU/CSU research team who compiled the data, which was then reviewed by the other authors of this study. In addition to the primary outcomes described below, a complete list of references provided by each team, scores for each, and review assignments are provided as Supporting Information: data (see “https://doi.org/10.18735/w9qc-9h39”).

### 2.5 Statistical analysis

The proportion of the time reviewers answered “yes” for our three research questions of whether the information was reported (Research Question 1); whether it was perceived to be reproducible (Research Question 2); and whether the occurrence differed between reported and reproducible metrics (Research Question 3) was summarized using logistic regression analysis. The R statistics package was utilized and the “logit” link function with a binomial generalized linear model was adopted.^29^, ^30^ Specifically, scoring consisted of the total number of “yes” and “no” results for each of the following factors: (1) the two reviewers, (2) the modeling categories, (3) the nine manuscripts, and (4) the “reported” or “reproducible” metrics. Output of the analyses yielded the proportion (0 to 1) of a “yes” answer for a given set of assumptions.

To determine the overall proportion of realizing a “yes” result for the reported (Research Question 1) and the reproducible (Research Question 2) metrics, the logistic regression analysis was performed assuming article and modeling category to be fixed effects while the reviewer was a random effect. Pairwise comparisons across the modeling categories were used to evaluate differences between the categories. This analysis was performed separately for the reported and reproducible metrics.

A pairwise comparison that included all categories was used to evaluate whether differences between the reported and reproducible metrics were statistically significant (Research Question 3). Results were evaluated as an interaction between the modeling categories and the reported or reproducible metric while the manuscripts were assumed to be a fixed effect and the reviewer was modeled as a random effect. Pairwise comparisons were also conducted to evaluate whether the reported and reproducible metrics were higher or lower for each individual category.

These analyses were performed for the major modeling categories (1–6 in “Evaluation criteria and outcome measures”) and separately for the subcategories in the “Model Structure” category (2.1−2.3). For all results, the proportions were reported with upper and lower 95% confidence intervals [shown in brackets] with *p* < 0.05 set as the threshold for statistical significance for pairwise comparisons. The compiled scores and the code used for statistical analysis were disseminated in a download package (see “https://doi.org/10.18735/w9qc-9h39”).

## 3 RESULTS

**Figure 4:**
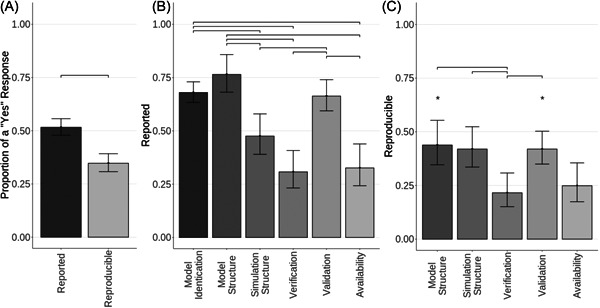
Category‐specific proportions of affirmative (i.e., “yes”) answers for (A) the reported and reproducible metrics across the major modeling categories, (B) the reported metric for the individual modeling categories, and (C) the reproducibility metric for individual modeling categories. 95% confidence intervals are included in each data set and designated with whiskers. The horizontal lines indicate statistically significant differences between the columns while the stars in (C) show the modeling categories that were significantly lower in reproducibility versus the same category in (B). All significant differences were defined as *p* < 0.05.

**Figure 5:**
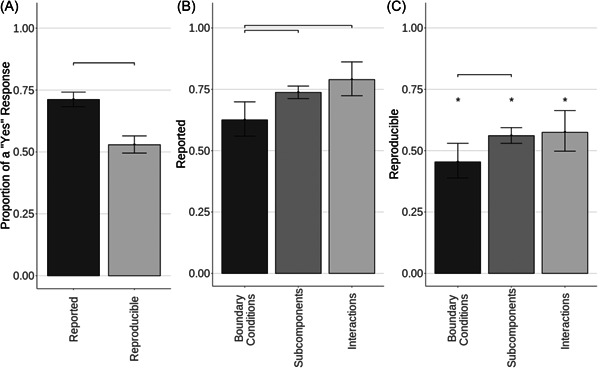
For the major “Model Structure” category (Figure 4), subcategory‐specific proportions of affirmative (i.e. “yes”) answers for (A) the reported and reproducible metrics across the modeling subcategories, (B) the reported metric for the individual modeling subcategories, and (C) the reproducibility metric for individual modeling subcategories. 95% confidence intervals are included in each data set and designated with whiskers. The horizontal lines indicate statistically significant differences between the columns while the stars in (C) show the modeling subcategories that were significantly lower in reproducibility versus the same category in (B). All significant differences were defined as *p* < 0.05.

Regarding our first research question, across all six major categories and for all nine studies, the proportion of the time reviewers answered “yes” for whether the information was reported was 0.52 [0.48, 0.56] (Figure 4A). For the subgroups under the Model Structure category (i.e., 2.1–2.3), the proportion of the time reviewers answered “yes” for reported was 0.71 [0.68, 0.74] (Figure 5A).

For the individual modeling categories, the proportion of the time reviewers answered “yes” for reported ranged from 0.31 [0.23, 0.41] (Verification) to 0.77 [0.68, 0.86] (Model Structure, Figure 4B). The Validation category's proportion of 0.66 [0.60, 0.74] was also relatively high while the Availability proportion was lower averaging 0.33 [0.24, 0.44]. The remaining Model Identification and Simulation Structure categories realized proportions of 0.68 [0.63, 0.73] and 0.48 [0.39, 0.58], respectively. Subcategory proportions for reported were 0.63 [0.56, 0.70] (Boundary Conditions), 0.74 [0.71, 0.76] (Subcomponents), and 0.79 [0.72, 0.86] (Interactions). Key findings for the categories in the reported metric were that Model Structure category was greater than the Simulation Structure (*p* = 0.0007), Verification (*p* < 0.0001), and Availability (*p* < 0.0001) categories (Figure 4B). The Verification category was less than Model Identification (*p* < 0.0001) and Validation (*p* < 0.0001) categories. Within the subcategories, the Boundary Conditions category was lower than the Subcomponents (*p* = 0.015) and Interactions (*p* = 0.003) categories.

Regarding our second research question, across all six major categories for all nine studies, the proportion the reviewers who perceived the studies to be reproducible was 0.35 [0.31, 0.39] (Figure 4A). For the subgroups under the Model Structure category (i.e., 2.1–2.3), the proportion of the time reviewers answered “yes” for reproducible was 0.53 [0.50, 0.56] (Figure 5A). Across the major categories, the reproducibility proportion ranged from 0.22 [0.15, 0.31] (Verification) to 0.44 [0.35, 0.55] (Model Structure, Figure 4C). For the subcategories, reproducibility proportions were 0.45 [0.39, 0.53] (Boundary Conditions), 0.56 [0.53, 0.59] (Subcomponents), and 0.58 [0.50, 0.66] (Interactions). Excluding Availability, the Verification category was lower than all other categories (*p* < 0.05, Figure 4C). Within the subcategories, the Boundary Conditions was lower than the modeling Subcomponents (*p* = 0.03, Figure 5C).

Regarding our third research question, across all major and submodeling categories, the reviewers answered “yes” for perceived reproducibility less than for reported (*p* < 0.05). The Model Structure and Validation categories were perceived to be less reproducible than reported (*p* < 0.05, Figure 4C). In all subcategories, the reproducibility metric was lower than the reported metric (*p* < 0.05, Figure 5C).

## 4 DISCUSSION

The aim of this study was to evaluate reporting and the potential for reproducibility across selected knee simulation studies that the research team collectively viewed as important contributions to the literature. Our main finding was that a representative subset of papers, which included work published by the coauthors of this manuscript, suffered from low reproducibility. Regarding our first research question, approximately half of the modeling components were reported across the major categories (i.e., the components of a model). Regarding our second research question, only about one‐third of modeling components were perceived to be reproducible (Figure 4A), with the exception of boundary conditions, specific components, and interactions (Figure 5C). Regarding our third research question, despite being reported, the Model Structure and Validation categories were perceived to be less reproducible (Figure 4C).

A cross‐section of studies was analyzed in this study, which allowed for an in‐depth analysis though generalizability of the conclusions may be limited. Alternative means of determining the cohort of studies were considered. These included using the number of citations and/or apparent experience of a given authorship list. Such approaches may have biased the cohort to older publications (for the number of citations) or toward the reporting practices of a narrower cross‐section of research teams. Interestingly, even studies published by the coauthors were perceived to have limitations in terms of reproducibility mitigating concerns for bias in favor of the authors' own work during the review process. Given the original directive of each team providing a list of manuscripts they deemed were valuable contributions due to their perceived impact and rigor, this cohort may not be representative of the wider literature. This analysis also did not consider whether each study was one publication within a series. A more in‐depth evaluation of related articles may have provided additional information on modeling features. Nevertheless, strengths of this study include providing a detailed analysis of a representative cohort of publications using a comprehensive assessment scheme. Additional strengths are use of a systematic approach to select representative and impactful work in the field. Our findings of low reproducibility corroborate reports in other fields^1^, ^2^, ^31^ and also supports the development of resources for dissemination of models.^11^, ^32^, ^33^, ^34^, ^35^ Moreover, this article provides a scalable framework to assess the reproducibility of computational modeling and simulation of musculoskeletal joints. This framework includes an assessment scheme, the raw data, and the code for statistical analysis enabling further expansion of the number of articles included in the analysis (see “https://doi.org/10.18735/w9qc-9h39”). Therefore, this article is an important first step to assessing reproducibility potential in computational modeling of the knee.

Model validation is often a primary focus of computational studies in the musculoskeletal literature.^36^, ^37^ Paralleling this emphasis, the reviewed studies fared relatively well in this category with two thirds of reviewers indicating validation procedures were reported (Figure 4B). While validation procedures were reported approximately two thirds of the time, their potential to be reproduced was lower with 40% of reviewers stating that these validation efforts were reproducible (Figure 4C). This indicates that validation is recognized as an important component of publication yet the potential to reproduce such studies is in question, which may ultimately impact the credibility of these models. It is worth noting that completeness of recording or the high potential for reproducibility does not warrant context‐of‐use relevant verification and validation and credibility assessment. That said, there were apparent deficiencies in both the reporting and reproducibility of verification and validation activities in the analyzed cohort (Figure 4B,C). While beyond the scope of this study, the legitimacy of these activities (e.g., a mesh convergence study, relevance of validation thresholds to model use context) was not assessed and should be considered a vital part of assessing a modeling and simulation study's credibility, beyond its potential to be reproduced.

Our findings on perceived potential for reproducibility are based on the subjective opinions of the five teams conducting the reviews rather than a direct attempt to reproduce any of the studies. Thus, the scoring of each article on their reproducibility potential depended on the author's direct experience in knee modeling. As such, the assessment of reproducibility was based on the extent to which the reviewer deemed the information in the study adequate to reproduce the modeling and simulation tasks given their comprehension of the information and their expertise. Arguably, if the documentation is perceived as incomplete, it would likely impact the study's reproducibility potential. Implicitly, the availability of data and code (if indicated in the manuscript) may have impacted the reviewer's assessment of reproducibility. Assuming computing resources were available to directly reproduce a given knee model, variations in software across the cohort of studies, which ranged from commercially available to lab‐specific, would have likely impeded such a task. Our findings revealed little consideration by the reviewers to the availability of software and judgments were made assuming reproduction attempts using their software of choice. Thus, the realized proportions could be considered conservative if reproducibility studies were undertaken. For example, in a repeatability study involving two of the authors in this study (A. E. and J. P. H.), multiple research teams reran an existing model using the same software and found that model predictions varied across teams even when using the same software.^38^ By disseminating the data and analysis in this study our hope is to inspire additional reviews, including to the presented cohort and possibly extending into additional focus areas and studies.

Our findings suggest that the reproducibility of computational modeling studies would likely improve if practices evolved to include dissemination of resources used in the modeling process, including data, code, and the models themselves. Across the reviewed studies, the proportion of reported and reproducible metrics regarding the Availability category were 0.33 [0.24, 0.44] and 0.25 [0.17, 0.36], respectively (Figure 4B,C). Posthoc analysis, however, revealed two of the nine reviewed manuscripts scored proportions greater than 0.5 in the reproducibility metric for the Availability category. This finding is likely because these two manuscripts included directions to download some or all of the modeling components.^9^, ^15^ This analysis was performed using the logistic regression analysis outlined in the Methods, but here the category was modeled as an interaction with the individual manuscripts and the reported or reproducible metric was a fixed effect (reviewer was a random effect). In contrast, the remaining seven manuscripts scored a maximum proportion of 0.13 for the reproducible metric in Availability. This bifurcation in score did not occur in any other modeling category.

Diversity in modeling methodologies may lead to variations in research findings, which may initiate inquiries that reveal the “hidden variables that underlie these differences.”^31^ Such independent reproducibility efforts may help identify modeling errors, which would prevent them from propagating through subsequent studies. Counter to this argument, the speed of development would be negatively impacted when studies are performed independently and without access to previous work.

Beyond reproducibility of preclinical modeling applications, to mature to the level of patient care, model predictions will also require evaluation against clinical data, which may also reveal deficiencies in a given approach. To mitigate investment in flawed models, alternative means to promote credibility such as benchmarking and/or certification have also been proposed.^11^ Benchmarking and/or certification by independent third parties—with limited public disclosure of research assets—may also provide an avenue to establish the reproducibility and credibility of modeling efforts that require the protection of proprietary data or modeling approaches.^39^ While this directly challenges complete reporting and the potential to be reproduced, it is one possible avenue to publish preclinical modeling studies that contain proprietary information. These efforts can focus on the compliance of the study to standards and best practices, with their outcomes reported along with the study. Other alternative strategies have also been discussed in the literature, that is, on perspectives for sharing of models and related assets.^11^ While such approaches increase the burden of development and dissemination, they also ground models using predetermined verification, validation, and/or quality metrics.

Our findings also suggest that reproducibility and reuse may require additional means of communicating modeling and simulation processes that supplement publication in archival journals. Standardization of modeling practices is one such means to enforce reproducibility and the completeness of reporting. While the assembled team and associated “KneeHub” project was created in part to address this need,^17^ there are additional noteworthy efforts. For example, many technical journals are evolving to include complementary tools that facilitate dissemination and viewing of data sets, results, and models.^32^, ^33^ The Physiome journal, in particular, was created “to encourage authors to make their models available in a manner that is reusable.”^35^ Physiome accomplishes this through dissemination using a predefined markup language and a curation process that tests model reproducibility before publication. Moreover, United States funding agencies require data sharing plans while the now expired NIH U01 “Predictive Multiscale Models for Biomedical, Biological, Behavioral, Environmental and Clinical Research” funding announcement enhanced this by requiring third‐party evaluation of model credibility.^40^, ^41^ Alternatively, reproducibility and reuse may dramatically improve when sharing of models and associated data becomes the norm. This will require the transformation of altruistic initiatives of model developers to a systemized approach promoted and monitored by sharing policies set by publishers and funding agencies. Beyond funding and publications, Findable, Accessible, Interoperable, and Reusable (i.e. “FAIR”) principles for data sharing have also been cited as an important component of modeling and simulation practices.^42^, ^43^, ^44^ While not directly addressing reproducibility and reusability, regulatory agencies, and in particular the Food and Drug Administration (FDA), have adopted guidelines on establishing credibility for modeling and simulation studies.^39^ Related resources include establishing the “10 rules for credible practice of modeling and simulation in healthcare,”^45^ the Physiome project^35^ and the Center for Reproducible Biomedical Modeling.^46^ Additional literature provides detailed discussion on the available resources for facilitating model reproducibility and reuse through model sharing.^11^

This current work contributes to a dialogue in the knee modeling community that parallels many other fields to facilitate the adoption of practices to improve reproducibility in preclinical science. Our findings of low reproducibility corroborate reports in other fields as evidenced by previous studies^1^, ^2^, ^31^ and the outlined resources for dissemination of models.^11^, ^32^, ^33^, ^34^, ^35^ Ongoing efforts, including our study, motivate the adoption of practices in biomechanical modeling and simulation of the knee joint that emphasize reproducibility. Such activities may facilitate clinical translation of these important simulation capabilities by supporting their credibility.

## AUTHOR CONTRIBUTIONS

**Halloran**: Substantial contributions to research design, or the acquisition, analysis or interpretation of data. Drafting the paper or revising it critically. Approval of the submitted and final versions. **Abdollahi Nohouji**: Drafting the paper or revising it critically. Approval of the submitted and final versions. **Hafez**: Substantial contributions to research design, or the acquisition, analysis, or interpretation of data. Drafting the paper or revising it critically. Approval of the submitted and final versions. **Besier**: Substantial contributions to research design, or the acquisition, analysis, or interpretation of data. Drafting the paper or revising it critically. Approval of the submitted and final versions. **Chokhandre**: Drafting the paper or revising it critically. Approval of the submitted and final versions. **Elmasry**: Drafting the paper or revising it critically. Approval of the submitted and final versions. **Hume**: Drafting the paper or revising it critically. Approval of the submitted and final versions. **Imhauser**: Substantial contributions to research design, or the acquisition, analysis, or interpretation of data. Drafting the paper or revising it critically. Approval of the submitted and final versions. **Rooks**: Drafting the paper or revising it critically. Approval of the submitted and final versions. **Schneider**: Drafting the paper or revising it critically. Approval of the submitted and final versions. **Schwartz**: Drafting the paper or revising it critically. Approval of the submitted and final versions. **Shelburne**: Substantial contributions to research design, or the acquisition, analysis, or interpretation of data. Drafting the paper or revising it critically. Approval of the submitted and final versions. **Zaylor**: Substantial contributions to research design, or the acquisition, analysis, or interpretation of data. Drafting the paper or revising it critically. Approval of the submitted and final versions. **Erdemir**: Substantial contributions to research design, or the acquisition, analysis, or interpretation of data. Drafting the paper or revising it critically. Approval of the submitted and final versions.

## CONFLICTS OF INTEREST

The authors declare no conflicts of interest.
